# Reverse Engineering Sparse Gene Regulatory Networks Using Cubature Kalman Filter and Compressed Sensing

**DOI:** 10.1155/2013/205763

**Published:** 2013-05-08

**Authors:** Amina Noor, Erchin Serpedin, Mohamed Nounou, Hazem Nounou

**Affiliations:** ^1^Department of Electrical and Computer Engineering, Texas A&M University, College Station, TX 77843-3128, USA; ^2^Chemical Engineering Department, Texas A&M University at Qatar, 253 Texas A&M Engineering Building, Education City, P.O. Box 23874, Doha, Qatar; ^3^Electrical Engineering Department, Texas A&M University at Qatar, 253 Texas A&M Engineering Building, Education City, P.O. Box 23874, Doha, Qatar

## Abstract

This paper proposes a novel algorithm for inferring gene regulatory networks which makes use of cubature Kalman filter (CKF) and Kalman filter (KF) techniques in conjunction with compressed sensing methods. The gene network is described using a state-space model. A nonlinear model for the evolution of gene expression is considered, while the gene expression data is assumed to follow a linear Gaussian model. The hidden states are estimated using CKF. The system parameters are modeled as a Gauss-Markov process and are estimated using compressed sensing-based KF. These parameters provide insight into the regulatory relations among the genes. The Cramér-Rao lower bound of the parameter estimates is calculated for the system model and used as a benchmark to assess the estimation accuracy. The proposed algorithm is evaluated rigorously using synthetic data in different scenarios which include different number of genes and varying number of sample points. In addition, the algorithm is tested on the DREAM4 *in silico* data sets as well as the *in vivo* data sets from IRMA network. The proposed algorithm shows superior performance in terms of accuracy, robustness, and scalability.

## 1. Introduction

Gene regulation is one of the most intriguing processes taking place in living cells. With hundreds of thousands of genes at their disposal, cells must decide which genes are to express at a particular time. As the cell development evolves, different needs and functions entail an efficient mechanism to turn the required genes on while leaving the others off. Cells can also activate new genes to respond effectively to environmental changes and perform specific roles. The knowledge of which gene triggers a particular genetic condition can help us ward off the potential harmful effects by switching that gene off. For instance, cancer can be controlled by deactivating the genes that cause it.

Gene expression is the process of generating functional gene products, for example, mRNA and protein. The level of gene functionality can be measured using microarrays or gene chips to produce the gene expression data [1]. More accurate estimation of gene expression is now possible using the RNA-Seq method. Intelligent use of such data can help improve our understanding of how the genes are interacting in a living organism [2–4]. Gene regulation is known to exhibit several modes; a couple of important ones include transcription regulation and posttranscription regulation [5]. While the theoretical applications of gene regulation are extremely promising, it requires a thorough understanding of this complex process. Different genes may cooperate to produce a particular reaction, while a gene may repress another gene as well. The potential benefits of gene regulation can only be reaped if a complete and accurate picture of genetic interactions is available. A network specifying different interconnections of genes can go a long way in understanding the gene regulation mechanism. The control and interaction of genes can be described through a *gene regulatory network*. Such a network depicts various interdependencies among genes where nodes of the network represent the genes, and the edges between them correspond to an interaction among them. The strength of these interactions represents the extent to which a gene is affected by other genes in the network. A key ingredient of this approach is an accurate and representative modeling of gene networks. Precise modeling of a regulatory network coupled with efficient inference and intervention algorithms can help in devising personalized medicines and cures for genetic diseases [6].

Various methods for gene network modeling have been proposed recently in the literature with varying degrees of sophistication [7–10]. These techniques can be broadly classified as static and dynamic modeling schemes. Static modeling includes the use of correlation, statistical independence for clustering [11–13], and information theoretic criteria [14–16]. On the other hand, dynamic models provide an insight into the temporal evolution of gene expressions and hence yield a more quantitative prediction on gene network behavior [17–20]. In order to incorporate the stochasticity of gene expressions, statistical techniques have been applied [13]. A rich literature is also available on the Bayesian modeling of gene networks [21–26]. Promoted in part by the Bayesian methods, the state-space approach is a popular technique to model the gene networks [27–33], whereby the hidden states can be estimated using the Kalman filter. In the case of nonlinear functions, the extended Kalman filter (EKF) and particle filter represent feasible approaches [33, 34]. However, the EKF relies on the first-order linear approximations of nonlinearities, while the particle filter may be computationally too complex. A comprehensive review of these methods can be found in [35].

In this paper, the gene network is modeled using a state-space approach, and the cubature Kalman filter (CKF) is used to estimate the hidden states of the nonlinear model [36, 37]. The gene expressions are assumed to evolve following a sigmoid squash function, whereas a linear function is considered for the expression data. The noise is assumed to be Gaussian for both the state evolution and gene expression measurements. As the gene network is assumed sparse, any simple mean square error minimization technique will not suffice for the estimation of static parameters. Therefore, a compressed sensing-based Kalman filter (CSKF) [38] is used in conjunction with CKF for reliable estimation of parameters. In case of statistical inference, it is essential to obtain some guarantees on the performance of estimators. In this regard, the Cramér-Rao lower bound (CRB) of the parameter estimates is used as a benchmarking index to assess the mean square error (MSE) performance of the proposed estimator which is evaluated here for a parameter vector. The performance of the proposed algorithm is tested on synthetically generated random Boolean networks in various scenarios. The algorithm is also tested using DREAM4 data sets and IRMA networks [39, 40].

The main contributions of this paper can be summarized as follows.

1. CKF is proposed for the estimation of states, and a compressed sensing-based Kalman filter is used for the estimation of system parameters. The genes are known to interact with few other genes only necessitating the use of sparsity constraint for more accurate estimation. The proposed algorithm carries out online estimation of parameters and is therefore computationally efficient and is particularly suitable for large gene networks.
2. The Cramér-Rao lower bound is calculated for the estimation of unknown parameters of the system. The performance of the proposed algorithm is compared to CRB. This comparison is significant as it shows room for improvement in the estimation of parameters.
3. The proposed algorithm is compared with the EKF algorithm. Using the false alarm errors, true connections, and Hamming distance as fidelity criteria, rigorous simulations are carried out to assess the performance of the algorithm with the increase in the number of samples. In addition, receiver operating characteristic (ROC) curves are plotted to evaluate the algorithms for different network sizes. It is observed that the proposed algorithm outperforms EKF in terms of accuracy and precision. The proposed algorithm is then applied to the DREAM4 10-gene and 100-gene data sets to assess the algorithm accuracy. The underlying gene network for the IRMA data sets is also inferred.

The rest of this paper is organized as follows.  Section 2 describes the underlying system model for the gene expressions. The proposed CKF algorithm in combination with CSKF for gene network inference is formulated in Section 3. The derivation of CRB is shown in Section 4, and the simulation results and their interpretation are presented in Section 5. Finally, conclusions are drawn in Section 6. 

## 2. System Model

Gene regulatory networks can be modeled as static or dynamical systems. In this work, state-space modeling is considered which is an instance of a dynamic modeling approach and can effectively cope with time variations. The states represent gene expressions, and their evolution in time, in general, can be expressed as

(1) $$\begin{array}{c} x_{k}=g\left(x_{k-1}\right)+w_{k}\;k=1,\ldots ,K, \end{array}$$

where *K* is the total number of data points available, **w**_*k*_ is assumed to be a zero-mean Gaussian random variable with covariance **Q**_*k*_ = *σ*_*w*_^2^ **I**, and the function *g*(·) represents the regulatory relationship between the genes and is generally nonlinear. The microarray data is a set of noisy observations and is commonly expressed as a linear Gaussian model [41]

(2) $$\begin{array}{c} y_{k}=h\left(x_{k}\right)+v_{k}, \end{array}$$

where **v**_*k*_ is Gaussian-distributed random variable with zero mean and covariance **S**_*k*_ = *σ*_*v*_^2^ **I** and incorporates the uncertainty in the microarray experiments. In order to capture the gene interactions effectively, the following nonlinear state evolution model is assumed [33, 34]:

(3) xk,n=∑m=1Nbnmf(xk−1,m)+wk,n,   k=1,…,K,  n=1,…,N,

where *N* is the total number of genes in the network and *f*(·) is the sigmoid squash function

(4) $$\begin{array}{c} f\left(x_{k-1,m}\right)=\frac{1}{1+e^{-x_{k-1,m}}}. \end{array}$$

This particular choice for the nonlinear function ensures that the conditional distribution of the states remains Gaussian [41]. The multiplicative constants *b*_*nm*_ quantify the positive or negative relations between various genes in the network. A positive value of  *b*_*nm*_ implies that the *m*th gene is activating the *n*th gene, whereas a negative value implies repression [34, 42]. The absolute value of these parameters indicates the strength of interaction.

The model given in (3) and (4) in the absence of any constraints may be unidentifiable and may result into overfitted solutions [43]. Assumptions on network structures are, therefore, necessary to obtain a connectivity matrix that agrees with the biological knowledge. In a gene regulatory network (GRN), the genes are known to interact with few other genes only. To this end, the coefficients *b*_*nm*_s are estimated using sparsity constraints, as explained in the next section.

A discrete linear Gaussian model for the microarray data is considered which can be expressed at the *k*th time instant as [41]

(5) $$\begin{array}{c} y_{k}=x_{k}+v_{k}. \end{array}$$

Stacking the unknown parameters together, the parameter vector to be estimated is

(6) $$\begin{array}{c} b≜\left[\phi_{1},\phi_{2},\ldots ,\phi_{N}\right], \end{array}$$

where *ϕ*_*n*_ = [*b*_*n*1_,…, *b*_*nN*_]. Plugging the values of states from (3) into (5), it follows that

(7) $$\begin{array}{c} y_{k}=R_{k}b+e_{k}, \end{array}$$

where

(8) $$\begin{array}{c} R_{k}≜\left[\begin{array}{cccc} {\tilde{f}}_{k} & 0 & 0 & 0\\ 0 & {\tilde{f}}_{k} & 0 & 0\\ 0 & 0 & \ddots & 0\\ 0 & 0 & 0 & {\tilde{f}}_{k} \end{array}\right], \end{array}$$

(9) $$\begin{array}{c} {\tilde{f}}_{k}≜\left[f\left(x_{k-1,1}\right)\cdots f\left(x_{k-1,N}\right)\right]. \end{array}$$

Thus, the gene network inference problem boils down to the estimation of system parameters **b** using the observations **y**_*k*_, where the effective noise **e**_*k*_ is the sum of system and observation noises. The next section describes the proposed inference algorithm for sparse networks. 

## 3. Method

**Figure 1:**
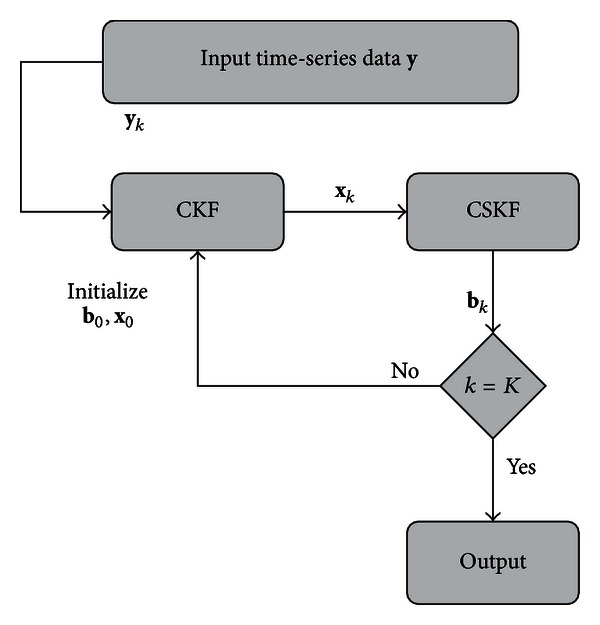
Block diagram of network inference methodology CKFS.

In this section, the methodology proposed to infer the system parameters in (3) is described. The proposed cubature Kalman filter with sparsity constraints (CKFS) approach is succinctly illustrated in Figure 1. The specific details of this algorithm are as next presented. 

### 3.1. Cubature Kalman Filter

Kalman filter is a Bayesian filter which provides the optimal solution to a general linear state space inference problem depicted by (1) and (2) and assumes a recursive *predictive-update* process. The underlying assumption of Gaussianity for the predictive and the likelihood densities simplifies the Kalman filter algorithm to a two-step process, consisting of prediction and update of the mean and covariance of the hidden states. However, the presence of nonlinear functions in the state and measurement equations requires calculation of multidimensional integrals of the form *nonlinear function  ×  Gaussian density* [36], which in general is computationally prohibitive. Several solutions to this problem have been proposed including the EKF, which linearizes the nonlinear function by taking its first-order Taylor approximation, and the unscented Kalman filter (UKF), which approximates the probability density function (PDF) using a nonlinear transformation of the random variable. Recently, a new approach, CKF, has been proposed which evaluates the integrals numerically using spherical-radial cubature rules [36].

The next two subsections briefly explain the working of Bayesian filtering and the CKF solution for the nonlinear multidimensional integrals.

#### 3.1.1. Time Update

Let the observations up to the time instant *k* be denoted by **d**_*k*_; that is, **d**_*k*_≜[**y**_1_^*T*^,…, **y**_*k*_^*T*^]^*T*^. In the prediction phase, also called the time update of the Bayesian filter, the mean and covariance of the Gaussian posterior density are computed as follows:

(10) $$\begin{array}{c} {\hat{x}}_{k\;|\;k-1}=E\left[f\left(x_{k-1}\right)\;|\;d_{k-1}\right],\\ P_{xx,k\;|\;k-1}=E\left[f\left(x_{k}\right)f^{T}\left(x_{k}\right)\right]-{\hat{x}}_{k\;|\;k-1}{\hat{x}}_{k\;|\;k-1}^{T}+Q_{k-1}, \end{array}$$

where *E* denotes the expectation operator and **x**_*k*−1_ is normally distributed with parameters $\left({\hat{x}}_{k-1|k-1},P_{xx,k-1|k-1}\right)$. The third equality is a consequence of the zero-mean nature of Gaussian noise **w** and its independence from **d**_*k*_. The estimates ${\hat{x}}_{k-1|k-1}$ and **P**_*xx*,*k*−1∣*k*−1_ are assumed to be available from the previous iteration. Here, **P**_*xx*,*k*∣*k*−1_ is an estimate of the error covariance matrix.

#### 3.1.2. Measurement Update

Since the measurement noise is also Gaussian, the likelihood density is given by $y_{k-1}|d_{k-1}:𝒩(z_{k-1};{\hat{y}}_{k|k-1},P_{xx,k|k-1})$. As the measurements become available at the *k*th time instant, the mean and covariance of the likelihood density are calculated as follows:

(11) $$\begin{array}{c} {\hat{y}}_{k\;|\;k-1}=E\left[y_{k}\;|\;d_{k-1}\right],\\ P_{yy,k\;|\;k-1}=E\left[x_{k}x_{k}^{T}\right]-{\hat{y}}_{k\;|\;k-1}{\hat{y}}_{k\;|\;k-1}^{T}+S_{k-1}. \end{array}$$

The updated posterior density, obtained from the conditional joint density of states, and the measurements can be expressed as

(12) ([xkTykT]Tdk−1)  ~𝒩((x^k ∣ k−1y^k ∣ k−1),(Pxx,k ∣ k−1Pxy,k ∣ k−1Pxy,k ∣ k−1TPyy,k ∣ k−1)),

where

(13) $$\begin{array}{c} P_{xy,k\;|\;k-1}=E\left[x_{k}x_{k}^{T}\right]-{\hat{x}}_{k\;|\;k-1}{\hat{y}}_{k\;|\;k-1}^{T} \end{array}$$

is the cross-covariance matrix between the states and the measurements. Hence, the states and the corresponding error covariance matrix are updated by calculating the innovation $z_{k}-{\hat{z}}_{k|k-1}$ and the Kalman gain **K**_*G*,*i*_

(14) $$\begin{array}{c} {\hat{x}}_{k\;|\;k}={\hat{x}}_{k\;|\;k-1}+K_{G,k}\left(y_{k}-{\hat{y}}_{k\;|\;k-1}\right),\\ P_{xx,k\;|\;k}=P_{xx,k\;|\;k-1}-K_{G,k}P_{yy,k\;|\;k-1}K_{G,k}^{T},\\ K_{G,k}=P_{xy,k\;|\;k-1}P_{yy,k\;|\;k-1}^{-1}. \end{array}$$

The next subsection briefly describes the computation of high-dimensional integrals present in the equations above.

#### 3.1.3. Computation of Integrals Using Spherical-Radial Cubature Points

In order to determine the expectations in (10), using a numerical integration method, a spherical-radial cubature rule is applied. This method calculates the cubature points **X**_*j*,*k*−1∣*k*−1_ as follows [36]:

(15) $$\begin{array}{c} X_{j,k-1\;|\;k-1}=U_{k-1\;|\;k-1}\zeta_{j}+{\hat{x}}_{k-1\;|\;k-1}, \end{array}$$

where $\zeta_{j}=\sqrt{\ell /2}{\left[1\right]}_{j}$, *j* = 1,…, *ℓ*, *ℓ* = 2*N* denotes the total number of cubature points and **U**_*k*−1∣*k*−1_ stands for the square root of the error covariance matrix; that is,

(16) $$\begin{array}{c} P_{xx,k-1\;|\;k-1}=U_{k-1\;|\;k-1}U_{k-1\;|\;k-1}^{T}. \end{array}$$

The cubature points are updated via the state equation

(17) $$\begin{array}{c} X_{j,k\;|\;k-1}^{*}=g\left(X_{j,k-1\;|\;k-1}\right). \end{array}$$

The propagated cubature points yield the state and error covariance estimates

(18) x^k ∣ k−1=1ℓ∑j=1ℓXj,k ∣ k−1∗,Pxx,k ∣ k−1=1ℓ∑j=1ℓXj,k ∣ k−1∗Xj,k ∣ k−1∗T −x^k ∣ k−1x^k ∣ k−1T+Qk−1.

The integrals in (11) and (14) can be evaluated in a similar manner. The next subsection explains the estimation of parameters in the system. 

### 3.2. Estimation of Sparse Parameters Using Kalman Filter

 The state estimates are obtained using the CKF as described in the previous subsection. In order to estimate the unknown parameters in the system model, one of the most commonly used methods involves stacking the parameters with the states and estimating them together. The estimation process performed in this manner is called *joint estimation*. Another method for the estimation of parameters consists of a two-step recursive process which is termed *dual estimation*. This process estimates the states in the first step, and with the assumption that states are known, parameters are estimated in the second step. These steps are repeated until the algorithm converges to the true values or until the amount of available observations is exhausted. This paper makes use of the latter technique.

The vector **b** as defined in (6) is assumed to be evolving as a Gauss-Markov model. As discussed previously, the states are assumed to be known at this step. The system evolution equations can therefore be expressed as

(19) $$\begin{array}{c} b_{k}=b_{k-1}+\eta_{k-1},\\ y_{k}=R_{k}b_{k}+e_{k}, \end{array}$$

where **η**_*k*_ stands for the i.i.d Gaussian noise and **R**_*k*_ is as defined in (8). It is observed that (19) is a system of linear equations with additive Gaussian noise, and therefore, the Kalman filter is the optimal choice for the estimation of parameter vector. The standard *predict* and *update* steps involved in Kalman filter are summarized as follows:

(20) $$\begin{array}{c} {\hat{b}}_{k\;|\;k-1}={\hat{b}}_{k-1\;|\;k-1}+\eta_{k},\\ P_{bb,k\;|\;k-1}=P_{bb,k-1\;|\;k-1}+\Sigma_{\eta_{k}},\\ u_{k}=y_{k}-R_{f_{k}}{\hat{b}}_{k},\\ K_{G}=P_{bb,k\;|\;k-1}R_{f_{k}}^{T}\left(R_{f_{k}}P_{bb,k\;|\;k-1}R_{f_{k}}^{T}+\sigma_{e}^{2}I^{-1}\right),\\ {\hat{b}}_{k\;|\;k}={\hat{b}}_{k\;|\;k-1}+K_{G}u_{k},\\ P_{bb,k\;|\;k}=\left(I-K_{G}R_{f_{k}}\right)P_{bb,k\;|\;k-1}, \end{array}$$

where **K**_*G*_ denotes the Kalman gain and **P** represents the error covariance matrix.

The Kalman filter algorithm is based on an *l*_2_-norm minimization criterion. As the gene networks are known to be highly sparse, the parameter vector is expected to have only a few nonzero values. A more accurate approach for estimating such a vector would be to introduce an additional constraint on its *l*_1_-norm which is the core idea in compressed sensing [38, 44]. Such an *l*_1_-norm constraint provides a unique solution to the underdetermined set of equations [45]. Therefore, instead of a simple *l*_2_ norm minimization, the following constrained optimization problem is considered:

(21) $$\begin{array}{c} \underset{{\hat{b}}_{k}}{\min}{||{\hat{b}}_{k}-b_{k}||}_{2}^{2}\;\text{s}.\text{t}.\;\;||{\hat{b}}_{k}||\leq \epsilon . \end{array}$$

The importance of this constraint can be judged by the fact that without it, the system would be rendered unidentifiable [43].

The problem (21) can be solved using a pseudomeasurement (PM) method which incorporates the inequality constraint (21) in the filtering process by assuming an artificial measurement ||**b**_*k*_||_1_ − *ϵ* = 0. This is concisely expressed as

(22) $$\begin{array}{c} 0=\overset{-}{R}{\hat{b}}_{k}-\epsilon ,\;{\overset{-}{R}}_{\tau }=\left[\mathrm{sign}\left({\hat{b}}_{\tau }\left(1\right)\right),\ldots ,\mathrm{sign}\left({\hat{b}}_{\tau }\left(N\right)\right)\right]. \end{array}$$

The value of the covariance matrix Σ_*ϵ*_ = *σ*_*ϵ*_^2^ **I** of the pseudonoise *ϵ* is selected in a similar manner as the process noise covariance in the EKF algorithm. However, it is found that large values of variances, that is, *σ*_*ϵ*_^2^ ≥ 100, prove sufficient in most cases [38]. Further details on selecting these parameters can be found in [38, 46]. The PM method solves (21) in a recursive manner for *K*_*τ*_ iterations using the following steps:

(23) $$\begin{array}{c} K_{G}^{\tau }=P_{\tau }{\overset{-}{R}}_{\tau }^{T}{\left({\overset{-}{R}}_{\tau }P_{\tau }{\overset{-}{R}}_{\tau }^{T}+\Sigma_{\epsilon }\right)}^{-1},\\ {\hat{b}}_{\tau +1}=\left(I-K_{G}^{\tau }{\overset{-}{R}}_{\tau }\right){\hat{b}}_{\tau },\\ P_{\tau +1}=\left(I-K_{G}^{\tau }{\overset{-}{R}}_{\tau }\right)P_{\tau }. \end{array}$$

At each *k*th time instant, **P**_*bb*,*k*∣*k*_ and ${\hat{b}}_{k|k}$ obtained from (20) are considered as initial values; that is, ${\hat{b}}^{1}={\hat{b}}_{k|k}$ and **P**_1_ = **P**_*bb*,*k*∣*k*_ which is the error covariance matrix. The value of *K*_*τ*_ is equal to the number of constraints, that is, the expected number of nonzero **b**_*mn*_s in the system. Possible ways for calculating *K*_*τ*_ include minimum description length (MDL) principle and Bayesian information criterion (BIC).

### 3.3. Inference Algorithm

The network inference algorithm is summarized in Algorithm 1. The algorithm consists of a recursive process which repeats itself for the number of observations present in the time-series data. For each time sample, the state estimate is obtained using the CKF, and the parameter estimate is obtained using the KF. Since the parameters are expected to be sparse, the estimates are then refined further using the CSKF algorithm. This iterative process results in a simple and accurate algorithm for gene network inference while considering a complex nonlinear model.

## 4. Cramér-Rao Bound

The performance of an estimator can be judged by comparing it with theoretical lower bounds proposed in parameter estimation theory. The CRB establishes a lower bound on the MSE of an unbiased estimator [47]. In particular, the CRB states that the covariance matrix of the estimator $\hat{b}$ is lower bounded by

(24) $$\begin{array}{c} 𝔼\left[\left(\hat{b}-b\right){\left(\hat{b}-b\right)}^{T}\right]⪰{\left[I\left(b\right)\right]}^{-1}, \end{array}$$

where the matrix inequality ⪰ is to be interpreted in the semidefinite sense and **I**(**b**) is the Fisher information matrix (FIM)

(25) $$\begin{array}{c} I\left(b\right)=𝔼\left[\left(\frac{\partial \ln f\left(y\;|\;b\right)}{\partial b}\right){\left(\frac{\partial \ln f\left(y\;|\;b\right)}{\partial b}\right)}^{T}\right]. \end{array}$$

The CRB for gene network inference can be calculated as follows. By stacking all the observations for *k* = 1,…, *K*, (7) can be written compactly in the matrix form

(26) $$\begin{array}{c} y=Rb+e, \end{array}$$

where **y** = [**y**_1_^*T*^,…,**y**_*K*_^*T*^]^*T*^, **R** = [**R**_1_^*T*^,…,**R**_*K*_^*T*^]^*T*^, and **e** = [**e**_1_^*T*^,…,**e**_*K*_^*T*^]^*T*^. The PDF *p*(**y** | **b**) is expressed as

(27) $$\begin{array}{c} p\left(y\;|\;b\right)=C\exp\left(-\frac{{\left(y-Rb\right)}^{T}\left(y-Rb\right)}{2\sigma_{e}^{2}}\right), \end{array}$$

where *C* is a constant. The derivative of ln⁡*p*(**y** | **b**) can be expressed as

(28) ∂ln⁡p(y ∣ b)∂b=−∂∂b[(y−Rb)T(y−Rb)σe2]=RTy−RTRbσe2.

It now follows that

(29) (∂ln⁡p(y ∣ b)∂b)(∂ln⁡p(y ∣ b)∂b)T  =RT(y−Rb)(y−Rb)TRσe4.

By taking the expectation of (29), the FIM in (25) is given by

(30) $$\begin{array}{c} I\left(b\right)=\frac{R^{T}R}{\sigma_{e}^{2}}. \end{array}$$

**Figure 2:**
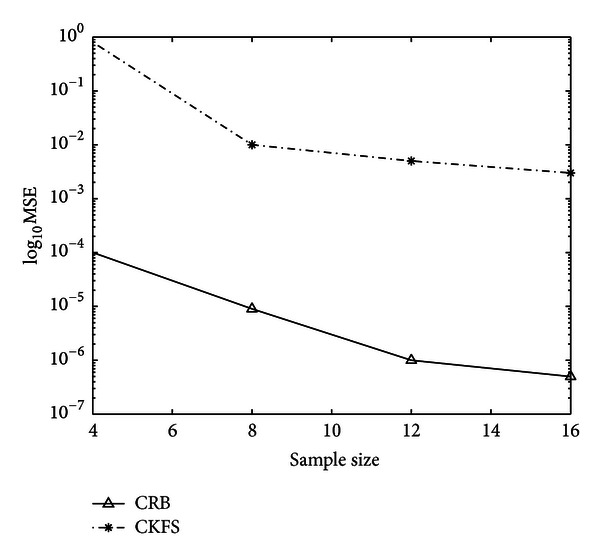
Cramér-Rao bound on the estimation of parameters. The MSE for one of the representative *θ* is shown here for a network consisting of 8 vertices.

The inverse of the FIM in (30) can be used to place a lower bound on the estimation error of the parameter vector **b**.  Figure 2 shows the comparison of MSE of CKFS algorithm with CRB as a function of number of samples *K* for one representative gene from the eight-gene network considered in Section 5.1. It is observed that the MSE of the estimated parameters decreases with increasing number of samples. 

## 5. Results and Discussion

The simulation results of the CKFS algorithm are discussed in this section. The performance is first tested on synthetic data obtained from randomly generated Boolean networks under various scenarios and performance metrics. The algorithm is then assessed on the DREAM4 networks and the IRMA network. 

### 5.1. Synthetic Data

 Time-series data is produced from randomly generated Boolean networks using the system model (3) and (5). Two scenarios are considered for this purpose.

**Figure 3:**
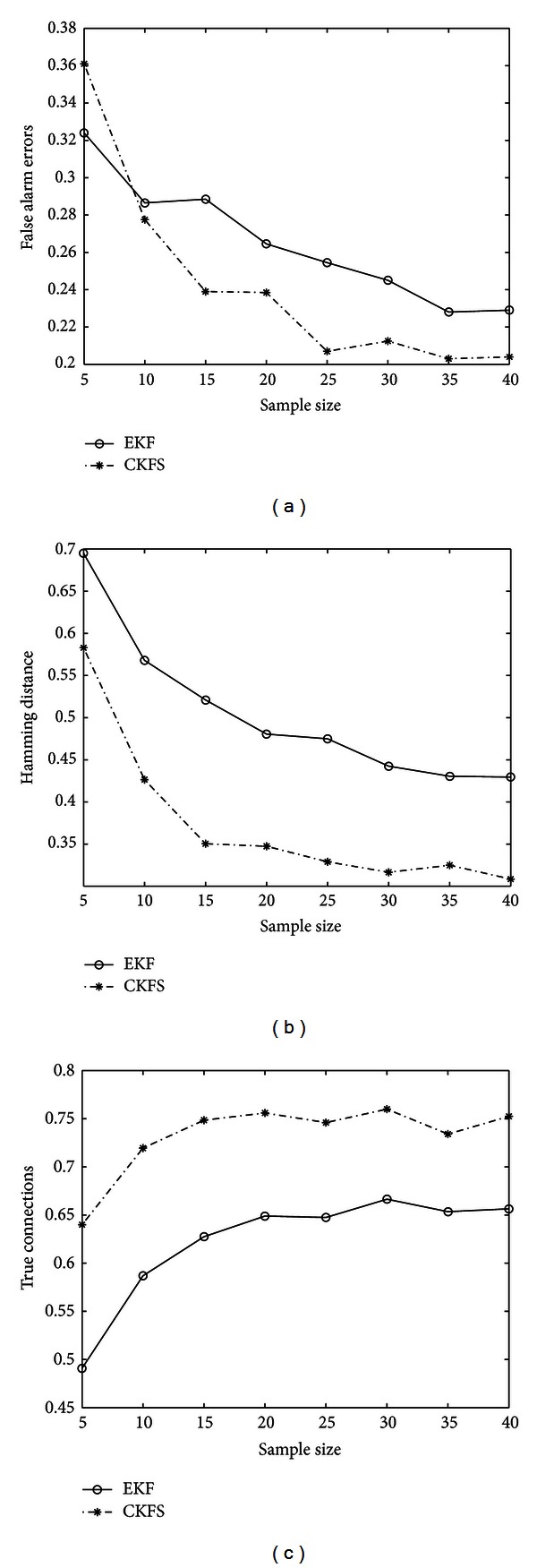
(a), (b), and (c) False alarm errors, Hamming distance, and true connections. The synthetic networks consist of 8 vertices and 20 edges. The metric is normalized over the number of edges. CKFS gives lower error and predicts more true connections with the increase in the sample size of data.

First, the comparison is performed by varying the number of sample size while keeping the network size fixed. The gene network consists of 8 genes and 20 vertices. In terms of network estimation, if the algorithm predicts an edge between two nodes which may not be present in reality, an error, referred to as *false alarm error* (F), is said to have occurred. Another situation is the indication of the absence of a vertex in the graph which in fact is present in the real network. This kind of error is termed *missed detection* (M). The summation of these two errors normalized over the total number of vertices in the network yields the *Hamming distance*. It is also important to consider the probability of predicting the true connections correctly which will be assessed by the *true connections* (T) metric. An algorithm with low Hamming distance and small false alarm error is particularly desirable as predicting an edge erroneously can be troublesome for biologists. True connections indicate the reliability of the predictions.  Figure 3 illustrates the performance of the CKFS algorithm and that of the EKF algorithm proposed in [34] in terms of the metrics described above. It is important to mention here that the same system model is assumed by both CKFS and EKF algorithms for the purpose of this simulation. These metrics are the same as those used in [15]. The variances of both the system and measurement noises, *σ*_*w*_^2^ and *σ*_*v*_^2^, respectively, are taken to be 10^−5^ in all the simulations and are assumed to be known. It is noticed that EKF has a slightly lower false alarm rate when the number of samples is small; however, as the number of samples increases, CKFS yields a lower false alarm error. The Hamming distance for CKFS is also smaller than EKF indicating lesser cumulative error. True connections show a consistent behavior for the two algorithms when the number of samples is increased where CKFS is able to predict connections more accurately. These experiments show the superiority of CKFS in terms of lower error rate.

**Figure 4:**
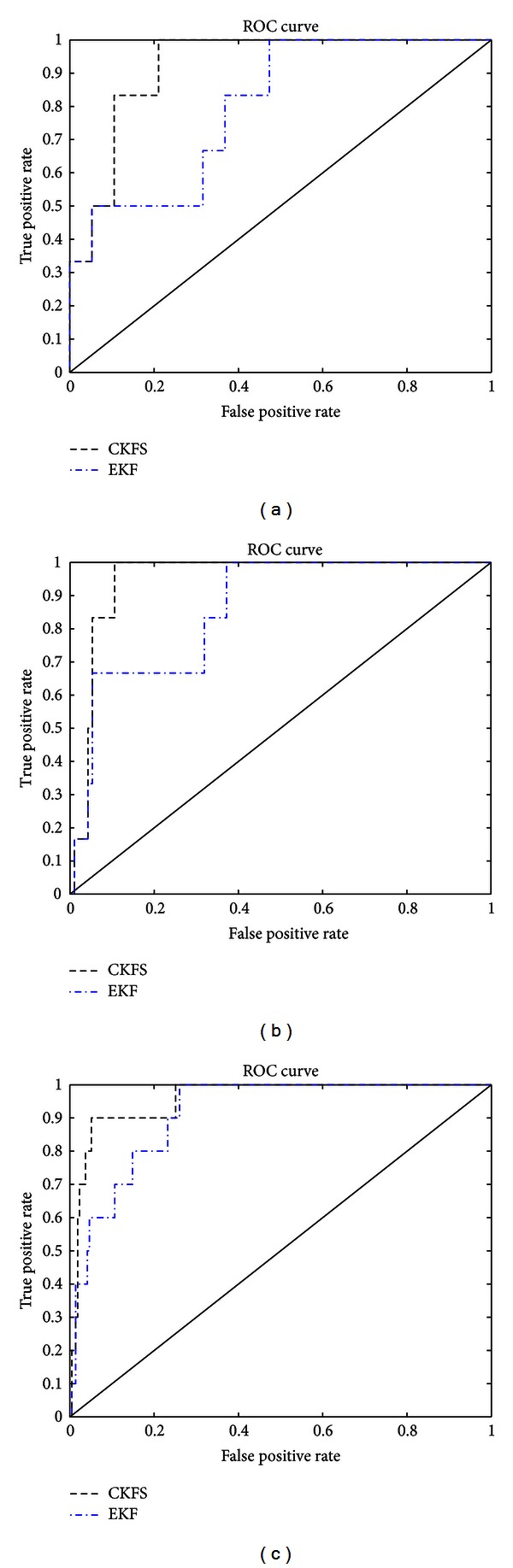
ROC curves for the performance of CKFS and EKF using synthetic data. (*N*, *E*, *K*) (a), (b), and (c) (5,10,20), (10,12,20), and (15,19,20). The area under the ROC curve for CKFS is more than that for EKF for various sized networks.

To obtain a more rigorous evaluation, the performance of algorithms is then compared in a scenario which considers the sample size to be fixed and assumes networks of different sizes. The receiver operating characteristic (ROC) curves are plotted as performance measures. A higher area under the ROC curve (AUROC) shows more true positives for a given false positive, and therefore, indicates better classification. The performance of CKFS(*N*, *E*, *K*) and EKF(*N*, *E*, *K*) is shown in Figure 4, where *N* stands for the number of nodes, *E* represents the number of edges, and *K* denotes the time points. It is observed that the CKFS exhibits superior performance than the EKF for networks of different sizes.

The complexity of the two algorithms is compared for synthetically generated networks with number of genes equal to 10, 20, 30, and 40. The sample size is kept to 50 time points for each of these networks, and the run time for EKF and CKFS algorithms is calculated as shown in Table 1. It is noted that EKF is faster for smaller network sizes, but as the network size increases, the run time gets much larger than that for CKFS. The main reason for this is that EKF [34] estimates the states and parameters by stacking them together which requires large-sized matrix multiplications at each iteration. The benefit associated with performing dual estimation, as in CKFS, is that the parameters are estimated separately from the states. Since the system is linear and one-to-one for parameters, inversion of much smaller matrices can be performed reducing the computational complexity of CKFS algorithm. CKFS is therefore particularly attractive for large-sized networks.

### 5.2. DREAM4 Gene Networks

Several *in silico* networks have been produced in order to benchmark the performance of gene network inference algorithms. dialogue on reverse engineering assessment and methods (DREAM) *in silico* networks serve as one of the popular methods used for this purpose [39, 48]. In this section, the performance of the CKFS algorithm is evaluated using the 10-gene and 100-gene networks released online by the DREAM4 challenge. Five networks are produced using the known GRNs of *Escherichia coli* and *Saccharomyces cerevisiae*. The data sets for each of 10-gene network consists of 21 data points for five different perturbations. The inference is performed by using all the perturbations. The 100-gene network consists of data sets for ten perturbations. AUROC and area under the precision-recall curve (AUPR) are calculated for the five networks of both the data sets and shown in Tables 2 and 3, respectively. The quantities, *precision* and *recall*, are defined as *P* = *T*/(*T* + *F*) and *R* = *T*/(*T* + *M*), respectively. For comparison purposes, the values of the two quantities for time-series network identification (TSNI) algorithm that exploits ordinary differential equations are also given [39]. The CKFS algorithm is found to perform significantly better than the TSNI algorithm.

### 5.3. IRMA Gene Network

**Figure 5:**
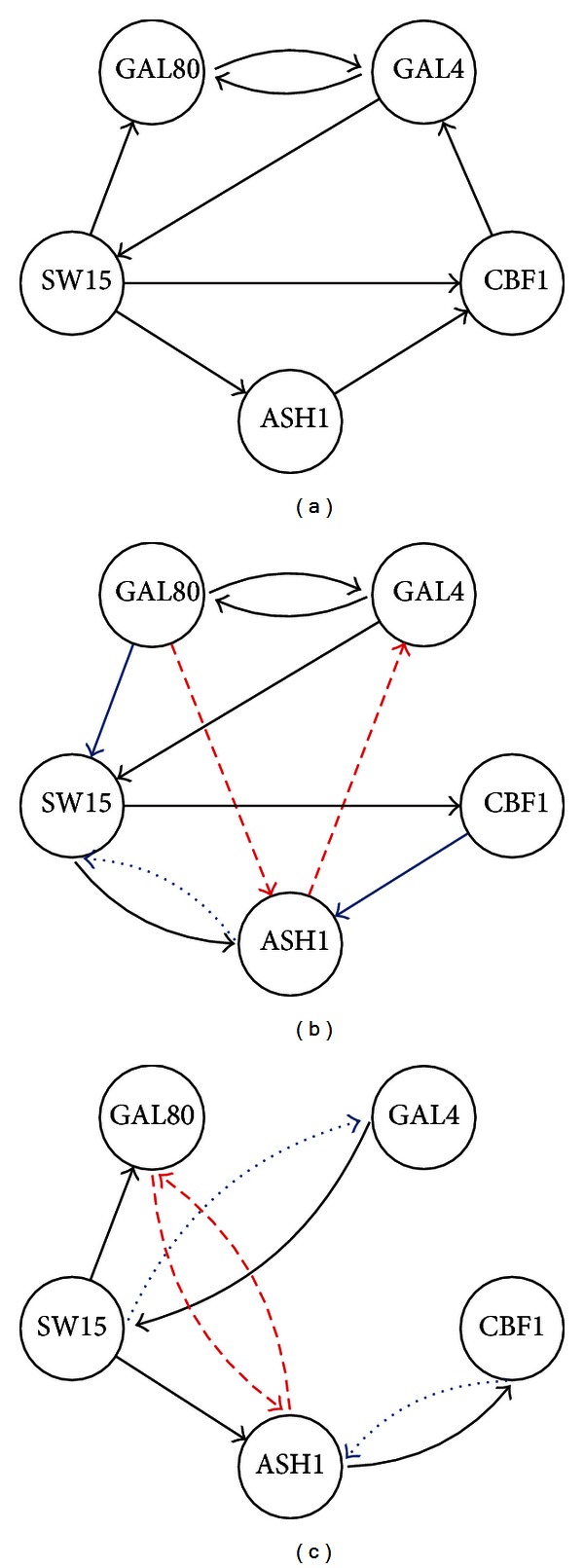
The inferred IRMA networks. (a), (b), and (c) Gold standard, inferred network using CKFS, and inferred network using ODE [39, 40]. Black arrows indicate true connections, blue arrows indicate the edges that are correct, but their directions are reversed, and red arrows indicate false positives.

In addition to synthetic data, it is imperative to test the algorithms using real biological data. In this subsection, the performance of the CKFS algorithm is assessed using the *in vivo* reverse-engineering and modeling assessment (IRMA) network [40]. This network consists of five genes. Galactose activates the gene expression in the network, whereas glucose deactivates it. The cells are grown in the presence of galactose and then switched to glucose to obtain the switch-off data which represents the expressive samples at 21 time points. The switch-on data consists of 16 sample points and is obtained by growing the cells in a glucose medium and then changing to galactose. The system and measurement noise variances for the CKFS are assumed to be identical as in the previous simulations.  Figure 5 shows the inferred network, the gold standard, and the network inferred using TSNI. It is observed that the CKFS algorithm succeeds to predict most of the interactions while giving lower false positives. 

## 6. Conclusions

This paper presents a novel algorithm for inferring gene regulatory networks from time-series data. Gene regulation is assumed to follow a nonlinear state evolution model. The parameters of the system, which indicate the inhibitory or excitatory relationships between the genes, are estimated using compressed sensing-based Kalman filtering. The sparsity constraint on the parameters is crucial because the genes are known to interact with few other genes only. The use of CKF and the dual estimation of states and parameters renders the algorithm computationally efficient. The performance of CKFS is evaluated for synthetic data for different network sizes as well as varying sample points. ROC curves, Hamming distance, and true positives are used for comparing the accuracy of inferred network with EKF. It is observed that CKFS outperforms the EKF algorithm. In addition, CKFS gives advantages over EKF in terms of smaller run time for large networks. The Cramér-Rao lower bound is also determined for the parameters of the model and compared with the MSE performance of the proposed algorithm. Assessment using DREAM4 10-gene and 100-gene networks and IRMA network data corroborates the superior performance of CKFS. Future research directions include incorporating the estimation of model order in the network inference algorithm.

## Figures and Tables

**Algorithm 1:**
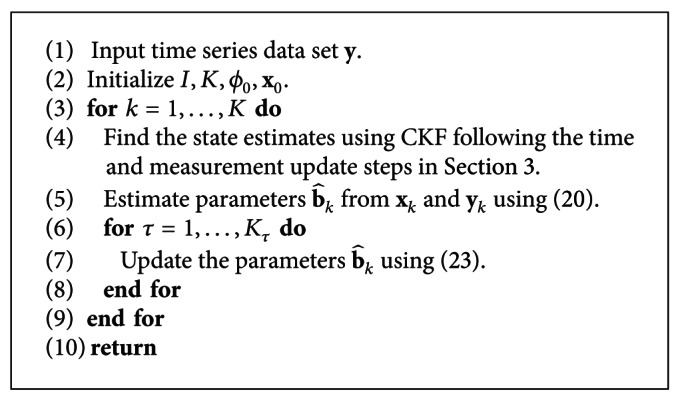
Network inference: CKFS.

**Table 1:** Run time in seconds for EKF and CKFS algorithms for varying network sizes for synthetically generated data. The number of sample points is fixed to 50.

Number of genes	10	20	30	40
EKF	0.16	1.9	16.5	84
CKFS	1.2	4.3	11.5	24.1

**Table 2:** Area under the ROC curve (AUROC) and area under the PR curve (AUPR) for DREAM4 10-gene networks for the five different networks.

Algorithm	Network 1	Network 2	Network 3	Network 4	Network 5
ODE [39]	0.62 (0.27)	0.63 (0.32)	0.58 (0.21)	0.63 (0.23)	0.68 (0.25)
CKFS	0.63 (0.40)	0.67 (0.50)	0.72 (0.50)	0.75 (0.49)	0.81 (0.42)
Random [39]	0.55 (0.18)	0.55 (0.19)	0.55 (0.17)	0.57 (0.17)	0.56 (0.16)

**Table 3:** Area under the ROC curve (AUROC) and area under the PR curve (AUPR) for DREAM4 100-gene networks for the five different networks.

Algorithm	Network 1	Network 2	Network 3	Network 4	Network 5
ODE [39]	0.55 (0.02)	0.55 (0.03)	0.60 (0.03)	0.54 (0.02)	0.59 (0.03)
CKFS	0.67 (0.13)	0.57 (0.08)	0.60 (0.10)	0.62 (0.10)	0.60 (0.07)
Random [39]	0.50 (0.002)	0.50 (0.002)	0.50 (0.002)	0.50 (0.002)	0.50 (0.002)
